# Thomas Lauder Brunton

**Published:** 1916-12

**Authors:** 


**Thomas Lauder Brunton,** M. D., LL.D., the distinguished pharmacologist, died in London, Sept. 16, aged 72.

